# The feasibility and accuracy of real-time intra-operative confocal tissue diagnosis in brain and spine cancer surgery

**DOI:** 10.1007/s10143-025-04083-y

**Published:** 2026-02-17

**Authors:** William S. Bolton, Oluwaseyi Adebola, Piravin K. Ramakrishnan, Dharsshini Reveendran, Vassili Crispi, Richard Digby, Rohitashwa Sinha, Arundhati Chakrabarty, Ryan K. Mathew

**Affiliations:** 1 Leeds Institute of Medical Research at St James’s, School of Medicine, University of Leeds, Leeds, LS9 7FT United Kingdom; 2 Department of Neurosurgery, Centre for Neurosciences, Leeds Teaching Hospitals NHS Trust, Leeds, LS1 3EX United Kingdom; 3 Department of Histopathology, Leeds Teaching Hospitals NHS Trust, Leeds, LS1 3EX United Kingdom

**Keywords:** Neurosurgery, Neurooncology, Histopathology, Intraoperative diagnosis, Confocal microscopy, Tumour

## Abstract

Real-time intra-operative brain tumour tissue analysis can reduce turnaround times and enable repeated sampling, enhancing diagnostic accuracy and guiding resection. We investigated the use of an ultra-fast confocal microscopy scanner (Histolog®, SamanTree Medical SA) for real-time brain tumour tissue diagnosis. This study aims to demonstrate Histolog®’s diagnostic accuracy in a cohort of brain or spinal cord tumour patients. A single-group, observational study included adult patients undergoing tissue biopsies or tumour debulking surgery. Multiple, freshly excised tissue samples were stained and imaged within 60 seconds using Histolog® alongside standard diagnostic methods. A Consultant Neuropathologist performed a blinded concordance analysis. Of 47 specimens, 94% produced interpretable images during surgery. Histolog® images enabled real-time tumour cell identification in all specimens and matched gold standard diagnoses in 68% of cases. Histolog® demonstrated rapid intraoperative imaging with diagnostic performance for tumour presence that was comparable to existing intraoperative methods in this small cohort, while exact histological concordance was lower (68%). Future studies will explore real-time margin zone analysis and machine learning for automatic diagnosis.

## Introduction

Histopathological tissue examination is the current gold standard diagnostic test in cancer surgery [1]. Methods like frozen section and squash cytology analysis are time consuming procedures often performed at remote locations from the operating room, and therefore have reduced practicality for rapid, repeat tissue sampling and examination that may be relevant for many different types of cancer surgery [2]. Precise intraoperative assessment of tissue is nonetheless extremely important. In neurosurgery for example, the intraoperative diagnosis of central nervous system (CNS) tumours by tissue smears or frozen section can provide guidance in modifying the surgical approach [3]. This can significantly increase total operative time with the associated risks of prolonged anaesthetic time and reduced theatre through-put.

Confocal microscopy, otherwise known as confocal laser scanning microscopy, is an optical imaging technique for enhancing optical resolution and contrast of a micrograph by utilising a spatial pinhole to block out out-of-focus light in image formation [4]. The Histolog® scanner developed by SamanTree Medical SA utilizes this technology to capture and display digital images of biopsied tissues on a mobile scanner, thereby enabling real-time intraoperative tissue analysis (https://www.samantree.com). It is currently being validated for use across many surgical specialties including urology and breast surgery. The Enclosure Study conducted at Canisius Wilhelmina Hospital in Netherlands evaluated the use of Histolog® scanner as an alternative to current standard practice in avoiding positive margins in prostate cancer surgery using NeuroSafe technique [5]. This study showed that using Histolog® scanner versus the current gold standard achieved similar performance but with 80% higher time efficiency.

During brain and spine cancer surgery, being unable to rapidly and repeatedly identify neoplastic areas limiting diagnostic confidence, reducing the precision of resection margins, and potentially constraining the extent of safe tumour removal. For example, patients stay asleep in theatre after biopsy until the lab confirms tumour has been hit (currently takes 60–90 minutes). And because of this timeframe, repeat sampling is discouraged and the most representative sample may be missed. Furthermore, current methods can be inaccurate at showing tumour margin detail, therefore a greater volume of residual tumour may remain, which is associated with earlier recurrence and worse survival, or unnecessary resection of normal brain may occur, increasing the risk of neurological deficit.

We aimed to investigate the feasibility and accuracy of Histolog® in brain and spine cancer surgery.

## Methods

### Study design, setting and population

An observational single group service evaluation was conducted in a tertiary UK brain tumour centre. As no change to patient care was made, no formal ethics was required given the nature of the service evaluation. The primary outcome was feasibility, defined as the proportion of intraoperative specimens for which the Histolog® scanner generated an interpretable image.

Secondary outcomes were: (i) diagnostic performance of Histolog® relative to paraffin-embedded histopathology, assessed as concordance at different diagnostic levels (tumour vs non-tumour; broad tumour class; exact histological diagnosis), and (ii) device usability as rated by theatre users on a modified usability questionnaire.

Adult patients undergoing either brain or spine biopsies and/or tumour debulking surgery were included and consent for tissue analysis for research purposes was obtained prior to surgery. Surgery was planned and performed as usual, and no alteration to the patient or surgical pathway was made. Inclusion criteria were: (i) adults (≥18 years), (ii) undergoing elective or emergency surgery for suspected primary or metastatic brain tumour, pituitary tumour, or spinal tumour, and (iii) in whom a fresh tissue specimen was sent for routine histopathological diagnosis. Exclusion criteria were: (i) patients <18 years, (ii) procedures in which no tumour specimen was obtained (e.g. aborted biopsies), and (iii) tissue not suitable for scanning (e.g. entirely cystic or haemorrhagic material without solid component). For this feasibility evaluation, we obtained one representative intraoperative specimen per patient immediately after the surgeon judged the sample adequate for diagnostic purposes. Tissue samples were analysed using the Histolog® scanner and sent to the histopathology lab for standard diagnostic processes for each included patient. The process of scanning using Histolog® does not alter the processes used by formal laboratory diagnosis. The digital images captured by Histolog® were stored and logged alongside basic demographic and clinical data such as the patient’s age, sex, tumour location and imaging characteristics. The images and information were then used by a consultant neuropathologist (who was blinded to the formal paraffin diagnosis) to attempt a diagnosis with Histolog® alone. The results of this were compared via concordance analysis to the results from the standard histopathological diagnosis with paraffin permanent section.

### Technology protocol

The Histolog Scanner (SamanTree Medical, Lausanne, Switzerland) is a CE-marked and FDA 510(k)-cleared wide field-of-view fluorescence confocal scanning microscope specifically engineered for real time high-resolution imaging of large biological tissues in clinical environments, including the operating room. To enhance tissue contrast, the Histolog Dip, a fluorescent dye solution based on acridine orange (SamanTree Medical, Lausanne, Switzerland), was applied directly to the specimen. The scanner generates high-resolution images using 488 nm excitation and collection of fluorescence above 500 nm. Images are produced without any post-processing with the fluorescence signal in white over a black background. A false colouration with a simple transposition into a purple signal over a white background is applied per default to facilitate readings by pathologists. It is operational within seconds of startup, with no need for user calibration or parameter adjustments. High-resolution surface images of the tissue are acquired in one minute per scan, allowing for immediate and detailed visualisation of tissue morphology down to the nuclear level. The Histolog® imaging system acquires a 4.8 × 3.6 cm (17 cm^2^) field of view in approximately one minute, using a fixed optical resolution of 2 μm, which permits visualisation of tissue architecture down to the cellular level. Tissue is briefly immersed in Histolog Dip™, a proprietary buffered Acridine Orange solution, which labels nucleic acids and selected proteins without compromising subsequent formal histopathology. The device captures fluorescence intensity as a white-on-black image; pseudo-colouring is applied via a simple intensity transposition that maps the white signal onto a purple-on-white palette to approximate toluidine-blue-like contrast for intraoperative interpretation. No additional image processing or computational enhancement is applied. This optical-sectioning fluorescence approach has been validated in published studies demonstrating preservation of downstream diagnostic quality and correlation with conventional H&E sections [6].

A group of surgical trainees and resident doctors were trained by the manufacturers to use the device correctly and standardise the technology protocol. The Histolog® scanner was wheeled from storage to the required theatre and could be used within the theatre itself. A new case file was created on the scanner with a study ID code that was used to pair the images with demographic and clinical information in the study database. Freshly excised tissue specimens were stained with the Acridine Orange for 10 seconds before being transferred to the scanning plate. The specimen was then sent for standard laboratory diagnosis. After use, the scanner shut down and cleaned with antiseptic wipes. The images were stored on the scanner and exported periodically to a secure shared drive that the neuropathologist could access.

### Data analysis

Quantitative data was tabulated and descriptive statistics generated using Microsoft Excel (Version 16.73). Feasibility was assessed because human brain tissue had not been examined with Histolog® before and the typical specimen size in neurosurgery is far smaller than other solid organ cancers. Feasibility was defined as the rate of failure to acquire an interpretable image from a specimen using the scanner. Quantitative concordance analysis between Histolog® results versus gold standard histopathology results was also carried out on Microsoft Excel. For each case, the neuropathologist’s Histolog® diagnosis was compared with the final paraffin-embedded histopathology report. Concordance was assessed at three levels:

1. Tumour presence (tumour present vs no tumour);
2. Broad tumour class (e.g. glioma, meningioma, metastasis, pituitary adenoma, lymphoma, schwannoma);
3. Exact histological diagnosis as reported in the final integrated WHO diagnosis (e.g. ‘glioblastoma, WHO grade 4’).

We report feasibility as the proportion of specimens with interpretable images, and diagnostic performance as the proportion of Histolog® diagnoses that matched the paraffin reference at each level of concordance.

A Modified Usability Scale was used to capture feedback from the technology users and inform the adoption of the technology into practice. The results from this were tabulated and summarised in Microsoft Excel.

## Results

This study presents data from the first use of Histolog® on primary human brain tissue globally.

### Patient demographics and tumour features

A total of *n*=50 patients were included in the final analysis. The majority of patients were male (*n*=34; 68%) with a mean age of 56 years. Most tumours were intra-axial (*n*=30; 60%). More detailed demographic and tumour information is summarised in Tables 1 and 2.

**Table 1:** Patient demographics and tumour characteristics

Variable	Value
Age (years) Mean (± standard deviation)	57 (14)
Sex M:F (*n*=; %)	34 (68%): 16 (32%)
Tumour location	-
Frontal (*n*=; %)	14 (28%)
Parietal (*n*=; %)	14 (28%)
Temporal (*n*=; %)	10 (20%)
Occipital (*n*=; %)	2 (4%)
Post fossa (*n*=; %)	0 (0%)
Pituitary (*n*=; %)	9 (18%)
Spinal (*n*=; %)	1 (2%)
Intra axial (*n*=; %)	30 (60%)
Extra axial (*n*=; %)	20 (40%)

**Table 2:** Tumour characteristics with Histolog® interpretation and formal diagnosis

Location of tumour	Intra/extra axial	Histolog alone	Histolog with clinical details	FS/Smear	Paraffin
Left temporal	Extra axial	Tumour likely metastasis	Tumour likely metastasis	nil	Meningioma
Left middle fossa skull base	Extra axial	No diagnosis possible	No diagnosis possible	nil	Epidermoid cyst
Left temporal/parietal	Intra axial	Glioma, likely High grade	Glioma, likely High grade	GBM	GBM
Pituitary fossa	Pituitary	Pituitary Adenoma	Pituitary Adenoma	nil	Pituitary adenoma
Right frontal parafalcine	Extra axial	Meningioma	Meningioma	nil	Meningioma
Left parafalcine	Extra axial	Meningioma	Meningioma	nil	Meningioma
Pituitary fossa	Pituitary	Pituitary Adenoma	Pituitary Adenoma	nil	Pituitary adenoma
Right parietal	Intra axial	Scan too thick for diagnosis	Scan too thick for diagnosis	GBM	GBM
Left frontal	Intra axial	Neoplasm, NOS	Neoplasm, NOS	Glioma, likely high grade	Anaplastic Oligodendroglioma WHO 3
Left parieto-occipital	Intra axial	High grade glioma	High grade glioma	High grade glioma	GBM
Left posterior frontal	Intra axial	Diffuse low grade glioma	Diffuse low grade glioma	Glioma, no high grade features	Oligodendroglioma WHO 2
Right parietal	Intra axial	Meningioma vs Glioma	Meningioma vs Glioma	High grade glioma	GBM
Left frontal	Extra axial	High grade glioma	High grade glioma	nil	Meningioma
Right temporal	Intra axial	Low grade glioma	Low grade glioma	Glioma, no high grade features	Astrocytoma, WHO 2
Right parietal	Intra axial	High grade glioma	High grade glioma	High grade glioma	GBM
Right temporal	Intra axial	Tumour Glioma	Tumour	Mainly necrotic, with focus of possible glioma	GBM
Right parietal	Intra axial	Tumour high grade	Tumour	Reactive or likely recurrent GBM	GBM
Right frontal	Intra axial	Tumour Lymphoma	Tumour	Metastatic carcinoma/melanoma, less likely glioma	Metastatic carcinoma
Left temporal	Extra axial	Tumour Meningioma	Tumour	Meningioma	Atypical meningioma
Left parietal	Intra axial	Tumour meningioma	Tumour	Metastatic carcinoma	Metastatic carcinoma
Right parietal	Intra axial	Tumour	Tumour	Mostly necrotic, Glioma	GBM
Right frontal	Intra axial	Tumour	Tumour	High grade glioma	Astrocytoma WHO 4
Left frontal	Intra axial	Tumour	Tumour	Lymphoma	Lymphoma
Right frontoparietal	Extra axial	Tumour meningioma	Tumour meningioma	Meningioma	Solitary Fibrous Tumour, WHO 2
Right frontoparietal	Intra axial	Faint staining, difficult to interpret	Faint staining, difficult to interpret	Glioma, likely high grade	GBM
Right frontoparietal	Intra axial	Tumour	Tumour	Glioma	Glioma, low grade
Skull base	Extra axial	Tumour	Tumour	Nil	Meningioma
Pituitary fossa	Pituitary	Pituitary adenoma	Pituitary adenoma	nil	Pituitary adenoma
Pituitary fossa	Pituitary	Pituitary adenoma	Pituitary adenoma	nil	Pituitary adenoma
Right frontal	Intra axial	Tumour	Tumour	Metastatic carcinoma	Metastatic adenocarcinoma
Right temporal	Intra axial	Tumour Meningioma Glioma	Tumour Meningioma Glioma	High grade glioma	GBM
Pituitary fossa	Pituitary	Pituitary adenoma	Pituitary adenoma	nil	Pituitary adenoma
Right frontal	Intra axial	Tumour	Tumour	Glioma, no high grade features	Astrocytoma WHO 2
Left frontal	Extra axial	Tumour -meningioma	Tumour -meningioma	Meningioma	Meningioma
Pituitary fossa	Pituitary	Pituitary adenoma	Pituitary adenoma	nil	Pituitary adenoma
Left temporal/insular	Intra axial	Tumour low-grade	Tumour low-grade	Diffuse glioma, no high grade features	Astrocytoma WHO 2
Left frontal	Extra axial.	Tumour -meningioma	Tumour -meningioma	Meningioma, no high grade features	Meningioma with brain invasion, WHO grade 2
L2/3 intradural tumour	Intradural extramedullary	Spindle cell tumour meningiomaschwannoma	Spindle cell tumour meningiomaschwannoma	nil	Schwannoma
Right temporal	Intra axial	Tumour	High grade tumour	Mostly reactive gliosis, few atypical cells	GBM
Left parietal	Intra axial	Tumour	Tumour	Lymphoma	Lymphoma
Right Parietal	Intra axial	Tumour Lymphoma	Lymphoma	Lymphoma	Lymphoma
Pituitary fossa	Pituitary	Pituitary adenoma	Pituitary adenoma	nil	Pituitary adenoma
Right angular gyrus	Intra axial	Tumour glioma	Tumour glioma	Glioma, no high grade features	Oligodendroglioma, WHO 2
Pituitary fossa	Pituitary	Pituitary adenoma	Pituitary adenoma	nil	Pituitary adenoma
Left peri-rolandic	Extra axial	Tumour meningioma	Tumour meningioma	nil	Meningioma
Left occipital	Intra axial	Tumour High grade	High grade tumour	Metastatic carcinoma	Metastatic carcinoma
Left temporal	Intra axial	Tumour	Tumour	Dysembryoplastic Neuroepithelial Tumour; Pilocytic Astrocytoma; High grade glioma	GBM
Left temporal	Intra axial	Tumour High grade	High grade tumour	High grade glioma	GBM
Left parietal	Intra axial	Suspicious of tumour	Suspicious of tumour	GBM	GBM
Pituitary fossa	Pituitary	Pituitary adenoma	Pituitary adenoma	Pituitary adenoma	Pituitary adenoma

### Feasibility and accuracy via concordance analysis

*N*=47 (94%) specimens were able to produce images that were interpretable during live surgery. Reasons for being unable were either faint staining or the scan being too thick. Feasibility was considered a success at this stage. The two main user dependent processes to achieve high quality images were generous staining and ensuring a flat, air-free contact with the plate. The training required to achieve standard scanning from the users was minimal.

**Fig. 1:**
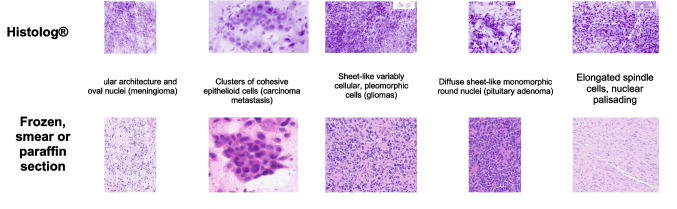
Examples of images produced via different methods. Figure consists of paired images from different tumours identified by the text labels with a brief visual description. Top row consists of example images produced by Histolog® and the bottom row presents corresponding examples from traditional frozen, smear or paraffin sections for visual qualitative comparison

When comparing the diagnosis results from Histolog® versus gold standard histopathology, 100% of the interpretable Histolog® images allowed the histopathologist to identify tumour cells during surgery itself in real-time. In *n*=34 cases (68%), the Histolog® images alone produced the exact diagnosis that gold standard histopathology resulted in. In the remaining interpretable cases, a diagnosis of ‘tumour’ was given. The images (examples in Fig. 1) produced demonstrated clusters of cohesive epitheliod cells in a case of metastatic carcinoma, sheet-like arrangement of variably cellular and pleomorphic cells in gliomas, diffuse sheet-like arrangement of monomorphic round nuclei in pituitary adenoma, elongated spindle cells in a schwannoma, and nodular architecture and oval nuclei in meningioma.

### Device usability

**Fig. 2:**
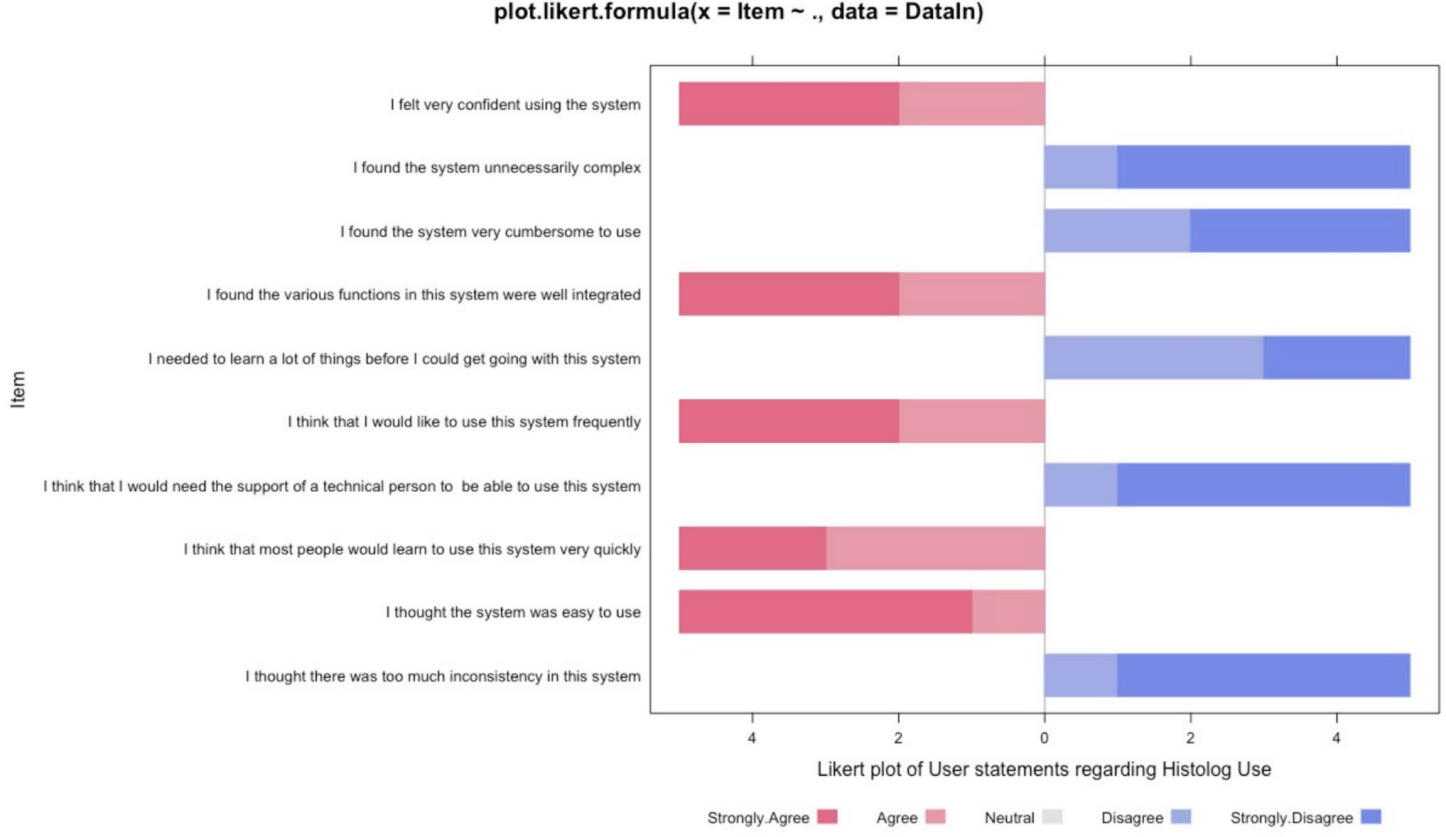
Modified usability scale for the Histolog® device use. Device usability scale statements and agreement/disagreement ratios presented with colour coded blocks

**Fig. 3:**
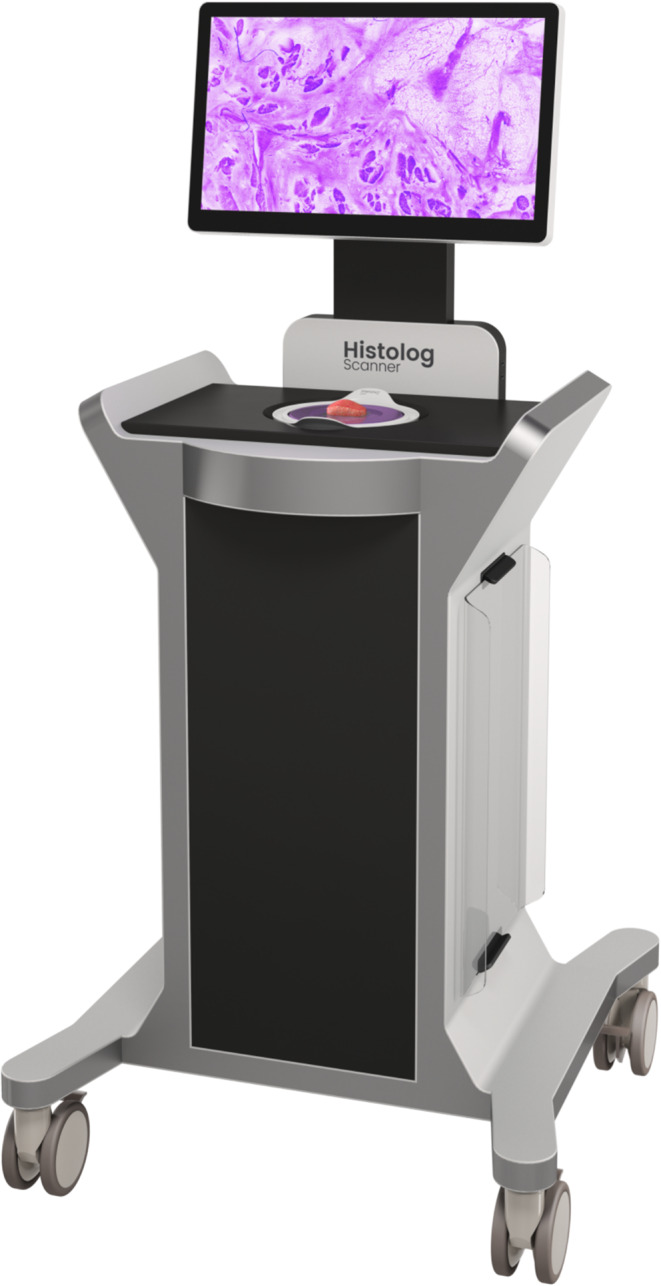
The Histolog ® Scanner

*N*=5 resident doctors or surgical trainees were trained to use the device during live surgery. Each received brief training and demonstrations (approx. 10–20 minutes) from the manufacturer before using the device for the study. They all completed a modified usability scale, and the results are displayed in Fig. 2. In summary, all device users felt the system was easy to use, that they could use it independent of a technical person, and that they could learn how to use it quickly. This suggests widespread adoption and use of the device is feasible. Fig. 3 displays an image of the device used.

## Discussion

In a world-first study investigating the feasibility and accuracy of this novel device in neurosurgery, our findings demonstrate that use of Histolog® to assist real-time intra-operative diagnosis in brain and spinal cancer surgery is feasible and that widespread adoption in theatre workflows may be achievable. The technology demonstrates a strong ability to identify tumour versus normal tissue, but formal laboratory diagnosis is still required to achieve higher rates of exact tumour type identification. These findings indicate that Histolog® is well suited for rapid confirmation of tumour cell presence and sampling adequacy, but it does not obviate the need for formal WHO-integrated CNS tumour diagnosis from paraffin-embedded tissue.

There is high confidence with using the images produced from Histolog® to identify if there is tumour present in the sample. This has significant implications on biopsy target confirmation and margin zone analysis during resection. Where histopathology services are located away from theatres, there is a time delay for the sample to reach an available histopathologist. This wasted time has implications for the patient themselves because of prolonged anaesthetic time and there is a health economics implication because less patients can be treated with the same amount of expensive theatre time. Full, integrated molecular diagnosis for CNS tumours will still be required, so the sample must still be sent to the laboratory for further tests. However, this technology can be used to confirm the target has been hit or if a margin zone is being reached.

Because of the speed of image acquisition in theatre itself, repeated sampling can be encouraged. This has implications for tumour debulking surgery because the process may allow the surgeon to make better informed resection decisions to improve the onco-functional balance. Real-time histopathological guidance using this technology currently relies on the availability a histopathologist who can view the images in real-time using the remote viewer capability in the product. Strategies to maximise the use of the technology in this way could include training surgeons themselves to recognise tumour versus normal tissue. This process could be augmented with the integration of artificial intelligence (AI) image analysis to help guide the human user further. Auto-recognition of tumour cells has been demonstrated in several tumour types [7]. In this way, surgeons could utilise real-time histopathological guidance during cancer surgery. It would be interesting to see how this guidance interplays with other forms of surgical guidance such as intra-operative fluoresce and radiological navigation.

The images produced by Histolog® were often inadequate to make a specific diagnosis and frozen section or smear testing would likely still be required. This may change as the use of the Histolog® images increased and the production quality and interpretation experience increase. With the current data, we cannot claim that this technology is able to replace the need for frozen section in CNS cancer surgery. The main positive impact of this technology is in the rapid turnaround times. For context, stimulated Raman histology (SRH) combined with deep-learning-based classifiers have achieved intraoperative diagnostic accuracies in the mid-90% range versus paraffin histology, with image acquisition and prediction in just a few minutes [3]. Confocal laser endomicroscopy systems such as CONVIVO generally report accuracies above 85–90% for lesional tissue identification and tumour core characterisation, although performance at resection margins is more variable [8]. In comparison, our Histolog® cohort showed excellent sensitivity for tumour presence in interpretable samples but only 68% concordance for exact histological class, suggesting that its current role is as a rapid “tumour versus non-tumour” and sampling-adequacy tool rather than a full replacement for conventional intraoperative histology. Comparisons between Histolog® and other real-time intra-operative histology technologies such as CONVIVO from Zeiss (a confocal endomicroscope system) are needed to investigate if any intra-operative solution could replace frozen section intra-operatively [8].

A key strength of this study includes using a research design that confirms the feasibility in a broad range of CNS tumours, and this confirmation is a unique contribution for the device to literature. We identified similar diagnostic accuracy values to large case series in the literature from Martirosyan et al. and our findings support the safety and feasibility of the technology summarised in a 2019 review [9, 10]. The present study builds on existing work in several ways. Firstly, the Histolog® Scanner is a portable, theatre-compatible device that theatre staff can use during surgery with a standardised ultra-fast protocol compatible with real-time surgical workflows. Secondly, the data provide a prospective clinical validation of an immediately deployable device. Thirdly, the study also explored the usability of the device to facilitate planning the integration of the technology into a wide variety of surgical and cancer workflows. We intend to train more theatre staff to improve its adoption further. This study has several limitations. First, all Histolog® images were interpreted by a single experienced neuropathologist, which may overestimate performance and precludes assessment of inter-rater variability. Second, despite including 50 patients across a range of CNS tumours, numbers for individual tumour types are small, so tumour-specific accuracy estimates are imprecise. Third, image quality depended on user technique, particularly staining intensity and tissue mounting. Finally, we classified scans as ‘interpretable’ based on visual assessment of tissue morphology, which does not necessarily equate to being ‘diagnostically sufficient’ for exact tumour class or grade. Larger, multi-reader studies with formal image quality criteria are needed before widespread implementation. Further research is needed to define the health-economics and clinical outcomes of using the device.

In summary, real-time intraoperative confocal scanning with Histolog® during CNS tumour surgery is feasible and allows rapid confirmation of tumour cell presence without disrupting workflow. In the near term, the technology is best suited to support biopsy target confirmation and margin-zone sampling, particularly in settings where frozen section is slow or not available. Future work should include multi-centre studies with multiple neuropathologists, formal assessment of inter-rater agreement, integration with AI-based image analysis, and evaluation of health-economic and clinical outcomes (extent of resection, complication rates, theatre throughput). These steps will be essential to define the role of Histolog® within modern, multimodal neurosurgical guidance strategies.

## Data Availability

Anonymised data is available upon reasonable request via the corresponding author.
